# Occult breast cancer discovered due to a large mass in the axilla: a case report

**DOI:** 10.1093/jscr/rjaf137

**Published:** 2025-03-12

**Authors:** Ryusei Yoshino, Nanami Ujiie, Shunsuke Yasuda, Masahiro Kitada

**Affiliations:** Department of Thoracic Surgery and Breast Surgery, Asahikawa Medical University Hospital, 078-8510, 2-1-1-1 Midorigaoka Higashi, Asahikawa, Hokkaido 078-8510, Japan; Department of Thoracic Surgery and Breast Surgery, Asahikawa Medical University Hospital, 078-8510, 2-1-1-1 Midorigaoka Higashi, Asahikawa, Hokkaido 078-8510, Japan; Department of Thoracic Surgery and Breast Surgery, Asahikawa Medical University Hospital, 078-8510, 2-1-1-1 Midorigaoka Higashi, Asahikawa, Hokkaido 078-8510, Japan; Department of Thoracic Surgery and Breast Surgery, Asahikawa Medical University Hospital, 078-8510, 2-1-1-1 Midorigaoka Higashi, Asahikawa, Hokkaido 078-8510, Japan

**Keywords:** occult breast cancer, ductal carcinoma in situ, lymph node metastasis

## Abstract

Occult breast cancer (OBC) is a rare form of breast cancer that is detected due to regional lymph node metastasis in the axilla. The patient was a 77-year-old woman. Twenty-four years previously, she had undergone a breast-conserving surgery and axillary lymph node dissection for left breast cancer. Her chief complaint was a mass in the right axilla. The breast magnetic resonance imaging did not show any findings suggestive of malignancy in the right breast. Therefore, an axillary dissection was performed. The results of the histopathological examination diagnosed the patient as having axillary lymph node metastasis of breast cancer. A total mastectomy of the right breast was performed. The patient was diagnosed with ductal carcinoma in situ (DCIS). She is currently undergoing postoperative endocrine therapy with an aromatase inhibitor. This report also includes a discussion of the treatment of OBC and the literature on lymph node metastasis and surgery for DCIS.

## Introduction

Breast cancer is one of the most common malignant tumors in women, and early diagnosis and appropriate treatment are important for improving the prognosis of patients. Among these, occult breast cancer (OBC) is a rare form of breast cancer that is detected through lymph node metastasis or distant metastasis, while no clear tumor is found in the breast itself [1].

There are a wide variety of reports on prognosis and treatment outcomes, and the clinical significance of this condition is currently being investigated. Because there are no breast lesions, there is a possibility that the diagnosis will be delayed, and because lymph node metastasis is often already advanced at the time of discovery, OBC has a unique clinical position [2].

## Case presentation

The patient was a 77-year-old woman. She was 150 cm tall, weighed 61 kg, and had a BMI of 27.1 kg/m^2^. Her past medical history included left breast cancer, hypertension, dyslipidemia, uterine fibroids, and herniated intervertebral discs. Her family history included two brothers with malignant lymphoma.

Twenty-four years ago, she underwent a breast-conserving surgery and axillary lymph node dissection for left breast cancer. The pathological findings were pT1bpN0cM0, and she was positive for hormone receptors, so she underwent postoperative radiation therapy and hormone therapy, and her follow-up observation was completed. On physical examination, a soft, elastic mass measuring 5 cm in diameter was palpable in the right axilla. There was no evidence of enlarged lymph nodes in the neck, supraclavicular fossa, or groin. Blood tests showed normal tumor markers for CEA and CA15-3, and no abnormalities were found in the blood count or biochemistry.

**Figure 1:**
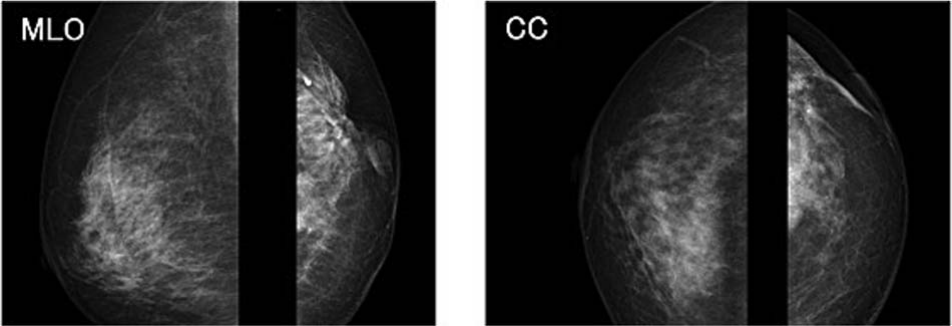
Chest radiograph (frontal view). No obvious abnormal findings were observed in the right breast.

**Figure 2:**
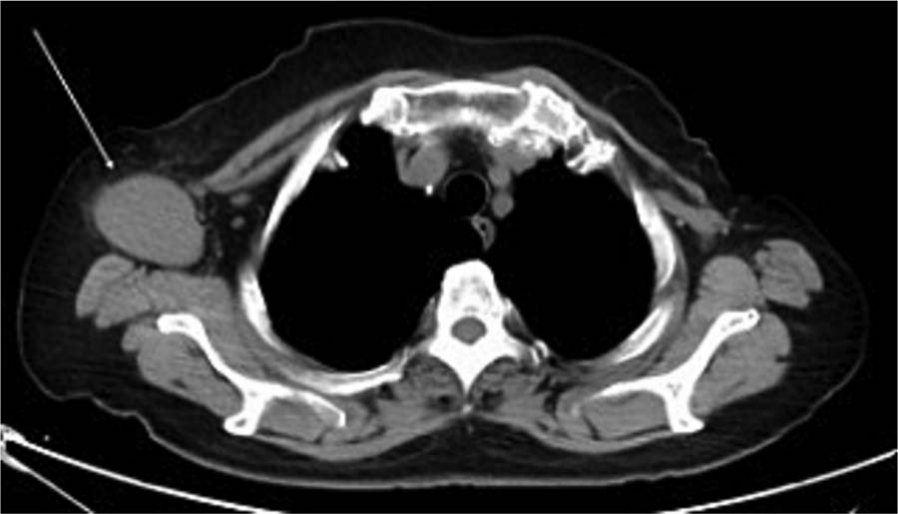
Chest computed tomography (CT) findings. A lymph node enlarged to 46 × 34 mm was observed in the right axilla.

**Figure 3:**
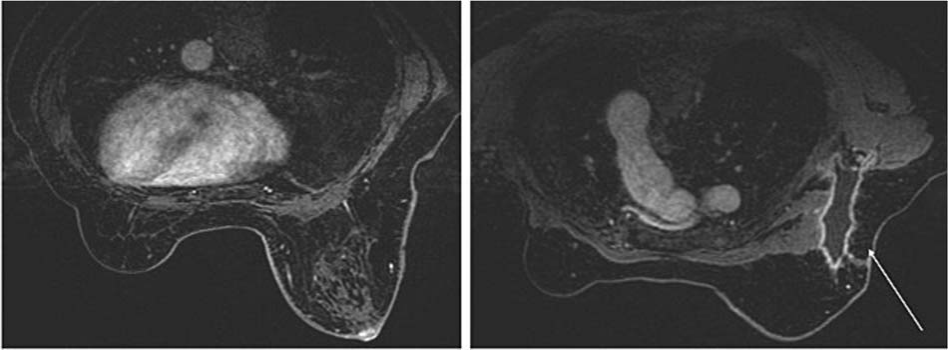
Breast magnetic resonance imaging (MRI) findings. No lesions were observed in the right breast, and a lymph node enlarged to 46 × 34 mm was observed in the right axilla.

The mammography examination did not reveal any obvious abnormal findings in the right breast (Fig. 1). The breast ultrasound examination also did not reveal any obvious abnormal findings in the breast. The chest computed tomography examination revealed a lymph node that had enlarged to 46 × 34 mm in the right axilla (Fig. 2). There were no enlarged lymph nodes in the mediastinum, hilar region or supraclavicular fossa, and there were no signs of distant metastasis. Magnetic resonance imaging (MRI) of the breast showed no lesions in the right breast, and a lymph node 46 × 34 mm in size was found enlarged in the right axilla (Fig. 3).

A fine-needle aspiration cytology was performed on the right axillary lymph node. Small atypical cells with relatively clear nucleoli were loosely aggregated to form sheet-like, scattered, or partially irregular papillary masses. There was no mucus production, and although the atypia was weak, the cells were found to be monotonous and epithelial-like in appearance, suggesting a carcinoma origin.

Based on the above test results, an axillary lymph node dissection was performed on the right axillary lymph nodes up to level III for the purpose of a definitive diagnosis, with OBC also in mind. The histopathological examination findings were as follows: metastatic carcinoma, histological grade II, (3 + 3 + 1 = 6), estrogen receptor (ER) (+), progesterone receptor (PR) (−), human epidermal growth factor receptor 2 (HER2) (2+) (no amplification by fluorescence in situ hybridization, FISH), ki-67 40%, lymph node metastasis (1/17). The primary tumor was thought to be in the breast, so a right mastectomy was performed as an additional procedure. The pathological histological examination findings were: ductal carcinoma in situ (DCIS) of intermediate nuclear grade, 3 mm in greatest dimension, histological grade II (3 + 3 + 1 = 6), ER (+), PR (+), HER2 (2+) (no amplification by FISH), ki-67 3%, pT1cN1M0, and pStage IIA. Her postoperative course was favorable, and she was discharged from the hospital on the 6th day after surgery. She is currently taking AI drugs as postoperative adjuvant therapy, and one year on, there has been no recurrence or metastasis.

## Discussion

A OBC is defined as a condition in which a tumor cannot be identified in both breasts clinically, and the only sign is metastasis to regional lymph nodes such as the axilla or supraclavicular fossa, and in which breast cancer is strongly suspected pathologically but the primary lesion is unknown [2]. When cancer metastasis is found in the axillary lymph nodes, breast cancer is the primary site in over 80% of cases, and other primary sites include stomach, lung, and larynx cancer [3]. Axillary lymph node dissection is necessary as a local treatment, and there are three treatment options for the breast: mastectomy, irradiation only, and observation. In addition, for latent breast cancer in which there is no primary lesion in the breast, it is said that non-mastectomy may be chosen with great care, based on the premise of whole-breast irradiation [1].

From this case, it was useful to perform a mastectomy on a patient with OBC in order to determine the treatment plan. In this case, the primary lesion was in the right breast, and the histopathological examination findings were DCIS. It has been reported that the rate of lymph node metastasis is low, at <5.0%, in patients who are diagnosed with DCIS before surgery and undergo sentinel lymph node biopsy [4]. This rate indicates that cases involving invasive cancer in the final pathological diagnosis are significantly associated with sentinel lymph node metastasis. Since invasive ductal carcinoma is generally the type of cancer that shows axillary lymph node metastasis, the fact that the primary lesion was diagnosed as DCIS, as in this case, was a very important finding in determining the decision on postoperative adjuvant therapy. In addition, since there have been no reports of OBCs discovered with axillary lymph node metastasis as the main complaint and with a histological type of DCIS, we believe this is a valuable case report.

In addition, it is thought that the axillary lymph node dissection up to level III in this case was appropriate. For patients with clinically evident positive axillary lymph node metastasis, axillary lymph node dissection up to level II is recommended, and it should be limited to cases where there is a macroscopic lymph node metastasis at level II or where there is clear suspicion of level III lymph node metastasis during surgery [5, 6].

## Data Availability

Data sharing is not applicable to this study because no datasets were generated or analyzed.
